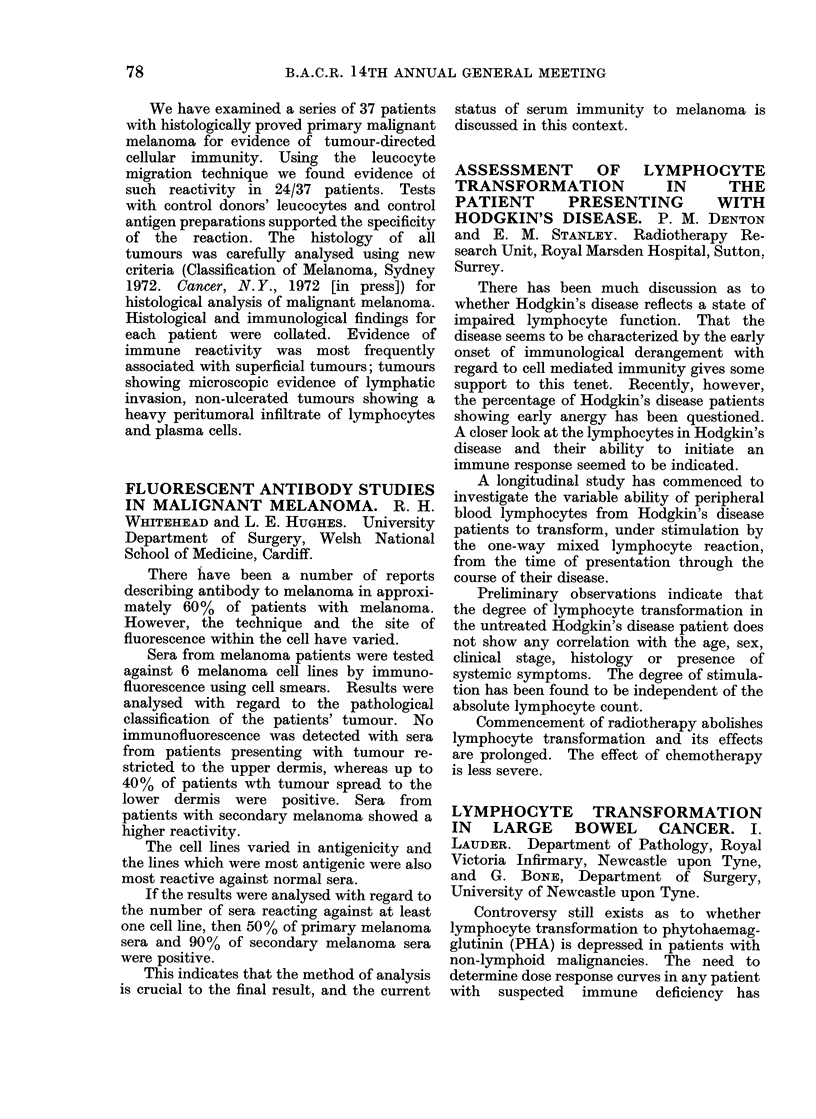# Assessment of lymphocyte transformation in the patient presenting with Hodgkin's disease.

**DOI:** 10.1038/bjc.1973.81

**Published:** 1973-07

**Authors:** P. M. Denton, E. M. Stanley


**ASSESSMENT OF LYMPHOCYTE TRANSFORMATION IN THE PATIENT PRESENTING WITH HODGKIN'S DISEASE.** P. M. DENTON and E. M. STANLEY. Radiotherapy Research Unit, Royal Marsden Hospital, Sutton, Surrey.

There has been much discussion as to whether Hodgkin's disease reflects a state of impaired lymphocyte function. That the disease seems to be characterized by the early onset of immunological derangement with regard to cell mediated immunity gives some support to this tenet. Recently, however, the percentage of Hodgkin's disease patients showing early anergy has been questioned. A closer look at the lymphocytes in Hodgkin's disease and their ability to initiate an immune response seemed to be indicated.

A longitudinal study has commenced to investigate the variable ability of peripheral blood lymphocytes from Hodgkin's disease patients to transform, under stimulation by the one-way mixed lymphocyte reaction, from the time of presentation through the course of their disease.

Preliminary observations indicate that the degree of lymphocyte transformation in the untreated Hodgkin's disease patient does not show any correlation with the age, sex, clinical stage, histology or presence of systemic symptoms. The degree of stimulation has been found to be independent of the absolute lymphocyte count.

Commencement of radiotherapy abolishes lymphocyte transformation and its effects are prolonged. The effect of chemotherapy is less severe.